# Noble UV protective agent for *Bacillus thuringiensis* based on a combination of graphene oxide and olive oil

**DOI:** 10.1038/s41598-017-11080-9

**Published:** 2017-09-08

**Authors:** Shahab Maghsoudi, Elham Jalali

**Affiliations:** 1 Department of Chemistry, Shahid Bahonar University of Kerman, Kerman, Iran P.O. Box, 76169-133 Kerman, Iran; 2 Young Researchers Society, Shahid Bahonar University of Kerman, P.O. Box, 76175-133 Kerman, Iran

## Abstract

The focus of this study is investigating the performance of graphene oxide (GO) in the protective effect of olive oil on *Bacillus thuringiensis* (*Bt*) after being exposed to UV radiations. Biological pesticides *Bt* subsp. *Kurstaki* is one of the most important biological control agents. We compared the protective effect of two UV protectant; GO and olive oil and also the combination of both, on the stability of the formulation of *Bt* after exposure to UV radiations. Spore viability was measured for protective effect and bioassay test was performed on the formulations of *Bt*. The combination of GO and olive oil revealed the highest viabilities of 50.62% after 96 h exposure to UV radiation, while viabilities of free spore, olive oil formulation and GO formulation were 32.54%, 37.19%,and 45.20%, respectively. The mortality of irradiated combination formulation on second-instar larvae *Ephestia Kuehniella* was 68.89%, while the same parameter for free spore, olive oil formulation and GO formulation were 40%, 46.66%,and 56%, respectively.

## Introduction

Nowadays the most chemical pesticides in agriculture are under stress to be eliminated from the market due to their harmful effects on the environment because people turn to biological pesticide use^1^. *Bacillus thuringiensis* (*Bt*) is the most common bacterial agent that is used for insect pest control^2^, which is a gram-positive and spore-forming bacteria. One of the disadvantages of biological pesticides is it’s low stability against natural factors such as UltraViolet (UV) radiation because the persistence of *Bt* crystal against insect pests are deactivated^3^. One way to prevail these disadvantages is to make the formulation of biopesticides by adding diverse UV protectants^4^. Nanotechnology is a novel technique which improves the stability of biopesticides against UV radiation by protecting the *B*.*thuringiensis* spores and crystals with the nanoparticle. Nanomaterials caused stable biological activity of the active agent in microbial pesticides and increased efficiency and the performance of their formulations.

GO, the two-dimensional (2D) nanoparticle with hydroxyl and epoxy bridge functional groups on the basal plane and carbonyl and carboxyl groups on the edges have obtained a quickly growing research interest^5–7^. These plentiful hydrophilic O-functional groups on the surfaces were beneficial in synthesizing in composites and maximizing the profit of the unique various properties^8^. In recent years graphene and graphene oxide (GO) were affirmed to be the ideal substrates for anchoring particles on their nanosheets’ surfaces, because of strong intermolecular bonding, high loading capacity, low surface energy and weak intermolecular bonding^9^.

Olive oil is a fat procured from the oil^10^. It’s authentication is based on the assessment of many parameters. Some of them are acidity, main fatty composition,and UV absorbance^11^. The UV protective effect of olive oil was determined by Jallouli^12^ as it has been shown, this is not the suitable property of olive oil.

In the present study, a nano-formulation of *B*. *thuringiensis* was prepared by Graphene oxide. The performance of graphene oxide in the protective effect of olive oil on *Bt* after exposure UV radiations was investigated. The effect of two UV protectant; GO and olive oil and the combined effect on the stability of formulations exposed to UV radiation were compared. The potency and spores viability of nano-formulation and non-nano-formulation were evaluated by the experimental methods in our laboratory.

## Results

### Assessment synthesis of GO

**Figure 1:**
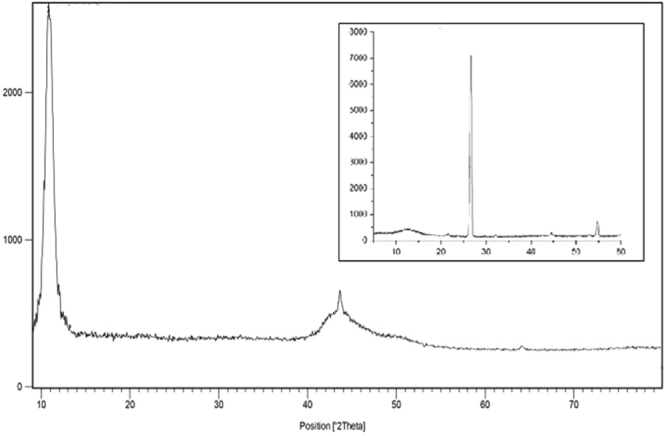
XRD spectra of GO and Graphite.

Figure 1 shows the XRD patterns of graphene oxide. Pristine graphite has a sharp diffraction peak at 26.34° which corresponds to a d-spacing of 0.334 nm^13^. This peak (002) has been disappeared after oxidation, while a peak at 10.79° was revealed which corresponds to a d-spacing of 0.85 nm and confirmed the successful preparation of GO^14^. An increased interlayer distance might be due to the formation of oxygen-containing functional groups, such as epoxy, hydroxyl, and carboxyl^15, 16^.

**Figure 2:**
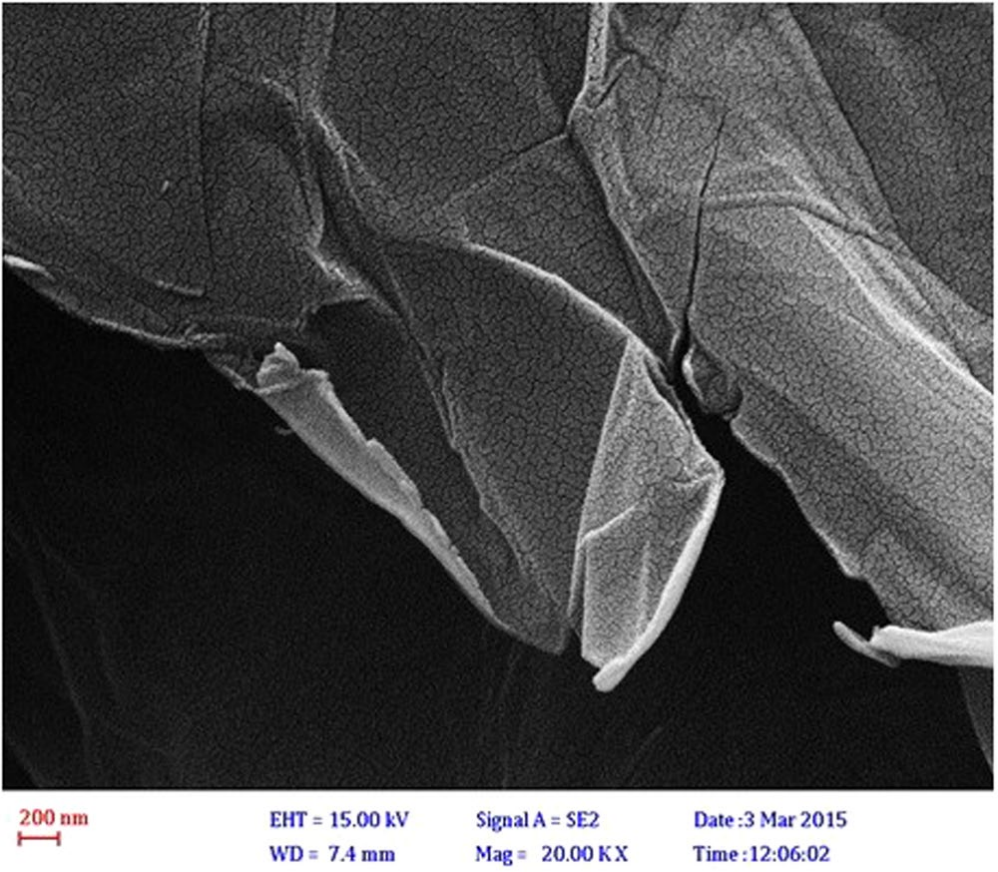
FESEM image of GO nanosheet.

The morphology and structure of GO nanosheets were studied using FE-SEM which has been illustrated in Fig. 2. The image is clearly indicative that GO nanosheets have layered structures with wrinkles and folds on the surface of GO.

**Figure 3:**
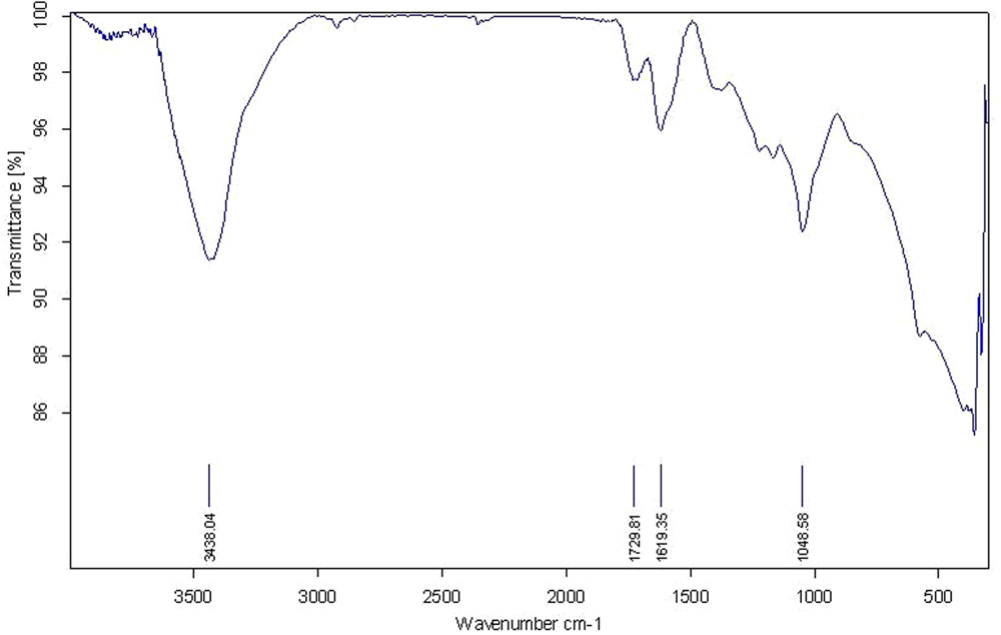
FT-IR spectrum of GO nanosheet.

Figure 3 shows FT-IR spectra of GO. The graph illustrates the O-H group stretching vibrations at 3438 cm^−1^. The absorption band at 1729 and 1619cm^−1^ corresponding to C=O stretching carboxylic functional groups and aromatic C=C bond, respectively. The band at 1048 cm^−1^ is assigned to the C–O stretching vibrations^17^.

### screening of UV protectant

**Figure 4:**
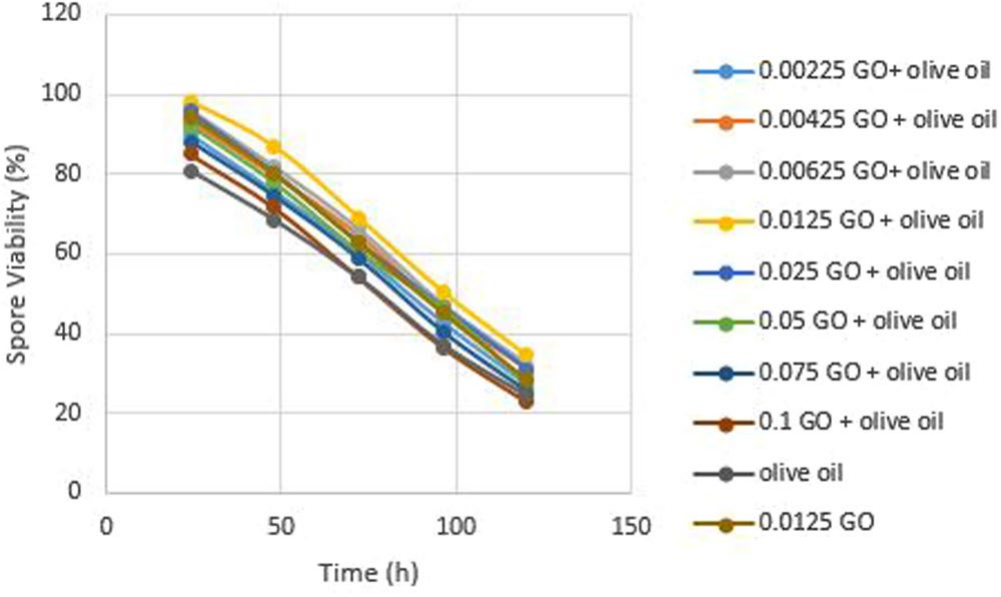
Effect of UV-A irradiation on spore viability of *Bt* mixture with different concentration of GO for different time exposure.

Photoinactivation of *Bt* which is one of the main environmental factors affecting its resistance has been investigated^18^. Evaluation of two UV protectants effect revealed that combination of olive oil and GO (0.0125% w/v) offered a significant *Bt* protection. Then, among the different tested GO concentrations, 0.0125% w/v was the most effective protecting formulation of *Bt* (Fig. 4) and for SPSS analysis this concentration was applied.

### Effect of UV radiations on spore count

In this study, the effect of UV-Radiation on the formulation of *Bt* was evaluated. As it can be seen in Table 1, the continuation of formulation’s exposure to UV radiations up to 96 h showed that spore count for free spore formulation of *Bt*, GO formulation, olive oil formulation and GO/olive oil formulation declined to 10.41 × 10^8^, 14.46 × 10^8^, 11.9 × 10^8^,and 16.2 × 10^8^ respectively. This is proportional to spore viability 32.54%, 45.20%, 37.19%, 50.62%. The results demonstrated that there was a significant difference between four *Bt* formulation at p = 0.05. Spores viability revealed that the GO/olive oil formulation has the highest protection of viability.

**Table 1:** The Bioassay and spore count for GO are tested at 0.025%. Bioassay was carried out on *Ephestia kuehniella*.

Treatment	Mortality	Spore viability (%)	cfu (10^8^ spores/ml)	Protection grade
Non irradiated free spore	100 ± 0.00^a^	100 ± 0.00^a^	32 ± 1.00^a^	
Irradiated free spore	40 ± 4.16^d^	32.54 ± 0.25^e^	10.41 ± 0.08^c^	C
Non irradiated GO formulation	100 ± 0.00^a^	100 ± 0.00^a^	32 ± 1.11^a^	
Irradiated GO formulation	56 ± 5.29^c^	45.20 ± 1.33^c^	14.46 ± 0.42^b^	B
Non irradiated olive oil formulation	100 ± 0.00^a^	100 ± 0.00^a^	32 ± 0.95^a^	
Irradiated olive oil formulation	46.66 ± 3.84^d^	37.19 ± 0.72^d^	11.90 ± 0.23^c^	C
Non irradiated combination formulation	100 ± 0.00^a^	100 ± 0.00^a^	32 ± 0.70^a^	
Irradiated combination formulation	68.89 ± 2.22^b^	50.62 ± 0.78^b^	16.20 ± 0.25^b^	A

A: >60% protection; B: 50–60% protection; C: <50% protection.

Note: Mean are the average of three replicates 45 larvae per in treatment, F = 86.228, df = 8, p = 0.0001. spore count for treatments was carried out using three replicates. F = 208.588, df = 7, p = 0.0001.

Means within the same column followed by a different letter are significant at p < 0.05, Duncan test.

The data in the table are mean ± SE.

### Effect of UV radiations on mortality against *E*. *kuehniella* larvae

**Figure 5:**
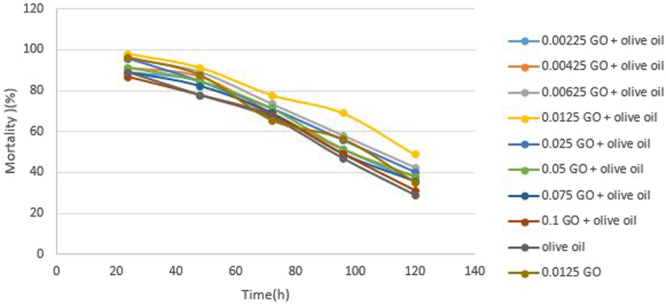
Effect of UV-A irradiation on mortality of larvae of *Bt* mixture with a different concentration of GO for different time exposure.

The larval mortality of irradiated GO formulation, olive oil formulation, GO/olive oil formulation and free spore of *Bt* after 96 h exposure to UV-A radiation were 56%, 46.66%, 68.89%,and 40%, respectively. There was a significant difference between treatment with GO formulation, GO/olive oil formulation (irradiated/non-irradiated), and irradiated free spore in mortality (p = 0.0001), while there was no significant difference between olive oil with free spore of *Bt*. The combination formulation of GO/olive oil in 0.0125% w/v showed the highest performance in mortality of larvae (Fig. 5). Poszgay *et al*.^19^ concluded that exposure of *Bt* to 40 h of UV radiations resulted in the loss of activity. Jallouli^12^ showed that no protection was observed in the case of olive oil and spore count was not significantly different from those when compared to the negative control exposed UV radiations also its effect has been under 50% protection. Our results of mortality have demonstrated that there was not a significant difference between olive oil formulation and free spore formulation at p = 0.05 and earlier works have been confirmed. On the other hand, there was a significant difference between GO formulation and formulation of GO/olive oil with free spore formulations of *Bt* (p = 0.0001). It is concluded that GO nanosheets effectively protected the *Bt* against UV radiations.

## Discussion

Since the 1990s of recent *Bt* strains and formulation, *Bt* products have advanced the development of *Bt* market by improvement in the insecticidal capabilities of the toxin. *Bt* has successfully replaced chemical pesticides for pest control in forestly^20^. *Bt* is a soil bacterium and it is the most widely used effective biological control agent in the world^21^. However, one of the main disadvantages is that the spores and insecticidal crystal proteins of *Bt* are vulnerable to degradation when exposed to ultraviolet radiation of sunlight under field condition^22–24^. Consequently, the utilization of *Bt* as a pesticide is more costly and finite compared to other alternatives due to the reduction of its biological activity.

It is generally believed that ultraviolet inactivation of the crystalline toxin is the significant source of the fast loss of *Bt*’s effectiveness^12^. Various materials and techniques have been checked as sunlight protectants to improve the *Bt* activity and prolong the efficacy of *Bt* pesticides in the environments such as the granular formulations, encapsulations,and addition of UV protectants^25–27^. Although this material can enhance the survival and persistence of efficacy of *Bt* formulations in the field they have some defects such as changeable, low shelf life, scant compatibility due to inhibition spore germination and microbial growth and wash off by the rain. So, for the choice of different adjuvants, it is needed to get necessary properties of *Bt* formulations^28, 29^.

Due to the rapid development of nanotechnology, it is crucial to understand how nanoparticles interact with living organism for biosafety reasons. The special chemical and physical properties of carbo-based nanomaterials define a broad range of options for practical applications and a range of studies have reported a positive impact of them on plant growth, exciting research on nanocarbon containing fertilizers^30–32^.

Antibacterial properties of GO were established to be dependent on the size of GO nanosheets; by enveloping bacterial cells, larger GO nanosheets can effectively isolate themselves from their environment and display stronger antibacterial activity as compared to small GO nanosheets^33^.

In this study, the effect of GO on the formulation of *Bt* with an attempt to increase its stability under UV-A radiation was examined. The GO was a UV absorbent as it was studied by Zhu^34^. It was shown that GO has an absorption at 335 nm and on the other hand, it has revealed a fluorescence about 440 nm. Therefore using GO in our combination formulation (GO + olive oil) resulted in UV-protectant (protection), and therefore the UV-protectant of olive oil was exclusively enhanced from 40% to 90%. This formulation has a competitive factor with other high protective agent such as molasses.

UV-A are measured to be the main part liable for inactivation of microbial pesticides. The sensitivity of *Bt* to UV radiations reduces its persistence in the environment. Diverse methods were used to increase the stability of biopesticides in field conditions.

The result showed that nanosheets GO could protect the formulation of *Bt* from UV-A and also it has an additive effect on the performance of olive oil as a protectant against UV radiation in *Bt*.

## Methods

### Materials

Natural graphite powders were purchased from Chem. Co. Fluka, sodium nitrate (NaNO_3_) potassium permanganate (KMnO_4_), concentrated sulfuric acid (H_2_SO_4_95%, d = 1.98 g/mL), Triton X-100, hydrochloric acid (HCl 37%, d = 1.18 g/mL), methanol and nutrient agar were purchased from Merck Chem. Co. (Germany). Hydrogen peroxide (H_2_O_2_, 30 wt. % aqueous solution) was purchased from Mojallali, Iran. Deionized (DI) Water used throughout all experiments and was purified with the Millipore system. Extra virgin olive oil was obtained from Guillen (Spain).

### Preparations of *B*. *thuringiensis*

*B*. *thuringiensis* subsp. Kurstaki KD-2 was obtained from the Iranian Research Institue of Plant Protection (Tehran, Iran). The suspension was centrifuged at 10000 rpm for 20 min and Spore Crystal Aggregate (SCA) was washed twice with distilled water and salt, that had been dried by lyophilisation and refrigerated at 4 °C^35, 36^.

### Synthesis of GO

GO was synthesized from the natural graphite powders by modified Hummer’s method^5, 37^. In brief, 2.5 g of graphite powder, 57.5 mL of H_2_SO_4_ and 1.25 g of NaNO_3_ were mixed under a vigorous stirring condition in an ice bath for 30 min. Then, 7.5 g of KMnO_4_ was added gradually in the portion under stirring for 1 h and the temperature was kept at 35 °C. The reaction mixture was cooled in an ice bath and 115 mL of water was added slowly to the solution, then 350 mL water and 15 mL H_2_O_2_ were added to the suspension. The suspension turned bright yellow and was washed three times with HCl (3%, 76 mL) and seven times with DI water until neutrality. The suspension in water laminated into GO nanosheets by a titanium-alloy solid probe ultrasonicator (20 kHz, 400 W, Topsonic, Iran). Finally, the GO was obtained as a gray powder after freeze- drying^38^.

### Preparation of nano-formulation by GO

A solution based on nano formulation by GO was prepared by dispersing 0.0125% of GO in water with ultrasonic vibration for 3 h. After, 0.20 g *Bt* and 0.05 g Triton X-100 was added and then shaked for 24 h in a dark place.

### Preparation of olive oil formulation

A mixture of 0.2 g *Bt* and Triton X-100 was mixed in 10 mL olive oil and then placed on a shaker set for 24 h in a dark place.

### Preparation of combination formulation by GO and olive oil

The different concentrations of GO solution (0, 0.00225, 0.00425, 0.00625, 0.0125, 0.025, 0.05, 0.075 and 0.1% w/v) were prepared by dispersed GO in water with ultrasonic vibration for 3 h, then 0.20 g of *Bt* and 0.05 g of Triton X-100 was added to the suspension. 10 mL of olive oil was added to the solution mentioned. The mixture was placed into a shaker set for 24 h in a dark place.

### Characterization

Powder X-ray diffraction (XRD) patterns were measured on a Phillips X’Pert PRO using filtered Cu Kα radiation (λ = 1.54178 Å) in the range of 2θ = 10–80°. The surface morphology of graphene oxide was characterized by field emission-scanning electron microscopy(FE-SEM, Sigma, Zeiss) with an acceleration voltage of 15 kV. Fourier transform infrared (FT-IR) spectra (4000–400 cm^−1^) were taken using a Tensor 27 spectrometer (Bruker, Saarbrucken, Germany).

### Assessment of nano-formulation

#### Assessment of spores viability after exposure to UV radiation

The diluted mixture from each of the formulations and the free spore formulation (30 mL) were exposed to UV-A radiation (385 nm) (Entela lamp model UVGL-25, 4 W). This was implemented in an open Petri dish at 15 cm distance from the lamp. Before designation of spore count, the amount of water evaporated in each sample was added to the formulation. The samples were then exposed to UV-A irradiation for 24, 48, 72, 96, and 120 h^12^. Spore counts were performed via serial dilution formulation spread in Petri dishes comprising nutrient agar and incubated at 28 °C for 24 h. Finally, the ratio of the number of the irradiated spores (nano or free formulation) to the number of the initial non-irradiated spores (nano or free spores), determined as spores viability, were calculated.

### Bioassay procedures

Second instars larvae of *E*. *kuehniella Zeller* was used for carrying out bioassay. Fifteen larvae were transferred to a sterile Petri dish which contained five peanut pieces that had been soaked in 30 mL of the formulation (irradiated and non-irradiated) under sterile conditions for 3 min and dried. Then incubated at 28 °C and 50–60% humidity^39^. A similar process was performed for non-nano-formulation and compared to nano-formulation and the control (peanut pieces soaked in sterile distilled water instead of bacterial suspension). Mortality was checked out every 24 h during 10 days and each treatment was repeated in triplicate.

### Data analysis

Analysis of Variance (ANOVA) was applied in Statistical Package for the Social Sciences (SPSS 1998). In order to determine the significant differences between treatments means, Duncan Test was applied.
